# Multimorbidity and quality of life in primary care: a systematic review

**DOI:** 10.1186/1477-7525-2-51

**Published:** 2004-09-20

**Authors:** Martin Fortin, Lise Lapointe, Catherine Hudon, Alain Vanasse, Antoine L Ntetu, Danielle Maltais

**Affiliations:** 1 Département de Médecine de famille, Université de Sherbrooke, 3001, 12^e ^Avenue Nord, Sherbrooke (Québec), J1H 5N4 Canada; 2 Département des Sciences humaines, Université du Québec à Chicoutimi, 555, Boulevard de l'Université, Chicoutimi (Québec), G7H 2B1 Canada

## Abstract

**Background:**

Many patients with several concurrent medical conditions (multimorbidity) are seen in the primary care setting. A thorough understanding of outcomes associated with multimorbidity would benefit primary care workers of all disciplines. The purpose of this systematic review was to clarify the relationship between the presence of multimorbidity and the quality of life (QOL) or health-related quality of life (HRQOL) of patients seen, or likely to be seen, in the primary care setting.

**Methods:**

Medline and Embase electronic databases were screened using the following search terms for the reference period 1990 to 2003: multimorbidity, comorbidity, chronic disease, and their spelling variations, along with quality of life and health-related quality of life. Only descriptive studies relevant to primary care were selected.

**Results:**

Of 753 articles screened, 108 were critically assessed for compliance with study inclusion and exclusion criteria. Thirty of these studies were ultimately selected for this review, including 7 in which the relationship between multimorbidity or comorbidity and QOL or HRQOL was the main outcome measure. Major limitations of these studies include the lack of a uniform definition for multimorbidity or comorbidity and the absence of assessment of disease severity. The use of self-reported diagnoses may also be a weakness. The frequent exclusion of psychiatric diagnoses and presence of potential confounding variables are other limitations. Nonetheless, we did find an inverse relationship between the number of medical conditions and QOL related to physical domains. For social and psychological dimensions of QOL, some studies reveal a similar inverse relationship in patients with 4 or more diagnoses.

**Conclusions:**

Our findings confirm the existence of an inverse relationship between multimorbidity or comorbidy and QOL. However, additional studies are needed to clarify this relationship, including the various dimensions of QOL affected. Those studies must employ a clear definition of multimorbidity or comorbidity and valid ways to measure these concepts in a primary care setting. Pursuit of this research will help to better understand the impact of chronic diseases on patients.

## Background

With technological advances and improvements in medical care and public health policy, an increasingly large number of patients survive medical conditions that used to be fatal. As a result of this phenomenon, and parallel to the aging of the population, a growing proportion of primary care patients presents with multiple coexisting medical conditions. From available data, it was estimated that 57 million Americans had multiple chronic conditions in 2000 and that this number will rise to 81 million by 2020 [1]. Epidemiological data from several countries support this estimate [2-8]. On average, patients aged 65 years and older present with 2.34 chronic medical conditions [7]. In fact, 50% of patients with a chronic disease have more than one condition [9].

The terms "comorbidity" and "multimorbidity" have been used to describe this phenomenon. Feinstein [10] originally described comorbidity as "any distinct additional entity that has existed or may occur during the clinical course of a patient who has the index disease under study." Kraemer [11] later referred to comorbidity in studying specific pairs of diseases. Van den Akker and colleagues [12] further refined both concepts, reserving the term "multimorbidity" to describe the co-occurrence of two or more *chronic *conditions; they also proposed some qualifiers to better classify the type of multimorbidity (simple, associative and causal). Unfortunately, much confusion still exists in the literature, where the 2 terms often seem to be used interchangeably. For the purpose of this paper, the term "multimorbidity" will be used according to Van den Akker and colleagues' definition and the focus will be solely on chronic diseases.

Previous reports on multimorbidity or comorbidity have documented that this phenomenon influences outcomes in many areas of health care [13-19]. Outcome measures that have been related to multimorbidity include mortality, length of hospital stay, and readmission. An association between disability and multimorbidity in elderly patients has also been described [14,20-22].

Quality of life (QOL) is an outcome measure that is increasingly being used to evaluate outcomes in clinical studies of patients with chronic diseases [23-26]. QOL represents a subjective concept, with a multidimensional perspective encompassing physical, emotional, and social functioning [27]. It is important to address QOL as it has been associated with health and social outcomes [28] which may contribute to the worsening of the course of the diseases. In research and the medical literature, there is little distinction between health-related quality of life (HRQOL) and overall QOL (the latter encompasses not only health-related factors but also many non medical phenomena such as employment, family relationships, and spirituality) [29]. In practice, the terms are often used interchangeably. Different evaluation scales have been proposed to measure QOL or HRQOL. Some focus on a specific disease [30,31], while others have wider applications (i.e., generic measurements) [32-34].

Little is known about the impact of multimorbidity on QOL of primary care patients [35], although this is where most patients receive their care. Thus, the purpose of this systematic review is to clarify the association between the presence of several concurrent medical conditions and the QOL or HRQOL of patients seen or likely to be seen in a primary care setting.

## Methods

### Data sources

**Figure 1:**
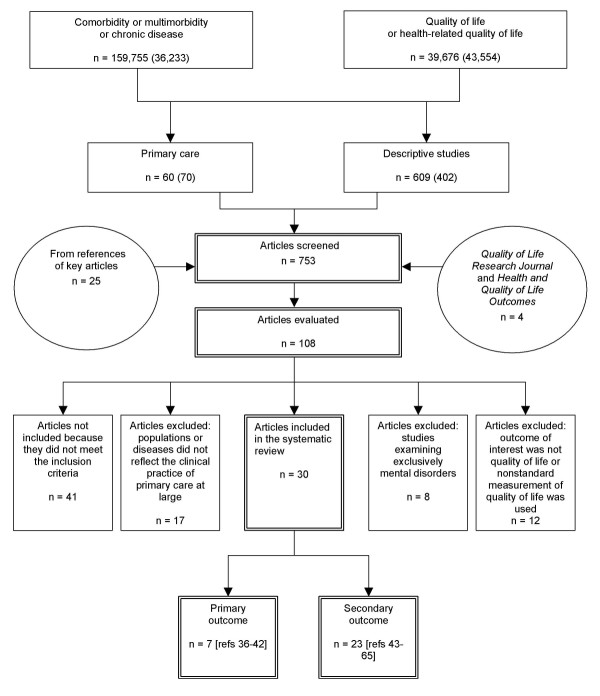
Selection of articles: Medline (Embase), years 1990–2003

For this review, we consulted Medline and Embase electronic databases for the reference period 1990 to 2003. Figure 1 illustrates the search strategy. Since the term "multimorbidity" does not have any equivalent in the thesaurus, databases were searched for the following terms: multimorbidity, comorbidity, and their spelling variations. The term "multimorbidity" was searched as a keyword, while "comorbidity" was searched as a Medical Subject Heading (MeSH). The term "chronic disease" was used to increase the sensitivity of the search. We also used the MeSH "quality of life" and the keyword "health-related quality of life" to help target pertinent literature.

To identify studies pertinent to the primary care setting, the following search terms were used: general practice, family practice, family medicine, family physician, and primary health care. A parallel strategy was used to identify all descriptive studies, regardless of the context of care, and the results were then combined. For the initial screening, the search was restricted to studies on human subjects, published in French or English. To be complete, we directly searched the *Quality of Life Research *and *Health and Quality of Life Outcomes *journals. We also screened references from key articles retrieved (hand searching).

### Study selection

One researcher (LL) performed the initial screening. Any ambiguous findings were discussed with the lead investigator (MF) and a consensus was reached.

#### Inclusion and exclusion criteria

For the purpose of this systematic review, we selected original, cross-sectional, and longitudinal descriptive studies that had evaluated the relationship between multimorbidity or comorbidity and QOL or HRQOL as the main outcome of interest. As stated earlier, we focused on the population of patients seen, or likely to be seen, in the primary care setting including members of the general population and residents of nursing homes and home healthcare facilities. We also selected original descriptive studies that had examined the relationship between multimorbidity or comorbidity and QOL or HRQOL as a secondary outcome.

Figure 1 shows our exclusion criteria. In keeping with our objectives, we did not include studies on specific diseases (e.g., acquired immunodeficiency disease) or populations unlikely to represent a large part of primary care practice. We also excluded any studies that did not address physical comorbidities, including those that exclusively examined mental disorders and associated mental comorbidities. Finally, we excluded studies in which the main outcome of interest was not QOL or HRQOL as well as those that used a nonstandard approach to measuring QOL or HRQOL.

#### Assessment of study quality

Before being included in the synthesis, the quality of each article selected was critically analyzed. For this assessment, we devised a scale in which points were assigned for study parameters indicative of good quality (e.g., well-defined populations, clear definitions, valid measures). Using this scale (Table 1), 2 researchers independently determined a global quality score for each article. The scores for each article were then compared and adjusted by consensus. To ensure adequate methodological quality, the cut-off score for an article to be included in the synthesis was 10 out of a maximum of 20 points.

**Table 1:** Evaluation criteria

Evaluation criteria for the studies identified in the literature search: 0, 1, or 2 points per criterion or subcriterion (maximum score = 20)
Criterion1: Originality
Original study (cross-sectional or longitudinal) with a clear objective
Criterion 2: Population studied
2a) Primary care or general population
2b) Well-defined control group or good variability of the independent variable in a regression model
2c) Characteristics of the groups are described, including those of nonrespondents, and do not lead to bias
Criterion 3: Definition
Clear definition of multimorbidity and valid measure
Criterion 4: Outcome
4a) Quality of life was the primary outcome measure
4b) Quality of life was evaluated with a validated scale
4c) Evaluation of quality of life was independent of the multimorbidity/comorbidity score (i.e., blind evaluation)
4d) Effects of the main confounding factors (e.g., age, gender) are presented and discussed
Criterion 5: Limitations
Authors comprehensively discussed the limitations of their study

## Synthesis or results

Figure 1 shows the number of articles found at each stage of the selection process. Of the 753 articles screened, 108 were evaluated according to the study's inclusion and exclusion criteria. We also assessed the quality of each study before selecting 30 for inclusion in the synthesis: 7 that had evaluated the relationship between multimorbidity or comorbidity and QOL as the main outcome (Table 2) and 23, as a secondary outcome.

### Quality of life as the main outcome measure

Of the 7 studies that featured QOL as a primary outcome [36-42], 5 had been conducted in European populations. We analyzed theses studies in detail. Quality scores for these studies ranged from 10 to 18 (out of a maximum of 20 points) and were highest in 2 studies from the Netherlands, one from the United States, and another study from Sweden. Table 2 presents a synthesis of the various studies.

All studies came to the same conclusion, namely that there is an inverse relationship between the number of medical conditions and QOL or HRQOL. This association may be affected by the patient's age or gender. Whereas multimorbidity mostly affects physical dimensions of QOL or HRQOL [36,37,41], data from one study suggest that social and psychological dimensions may be affected in patients with 4 or more diagnoses [40].

In each study, investigators relied on simple count of chronic diseases from a limited list to measure multimorbidity. The chronic conditions included in this list varied among the studies, and no attempt was ever made to assess or account for the severity of each condition. Furthermore, 5 of the 7 studies did not consider psychiatric comorbidity, either because the illnesses considered did not include psychiatric diagnoses or because patients presenting with psychiatric diagnoses were excluded from the QOL evaluation. In most cases, the diagnostic information was obtained by a questionnaire that was completed by a nurse or a doctor or sometimes self-administered. One study assessed comorbidity via chart review.

To measure QOL, a variety of scales were used. Most studies (5/7) used tools from the Medical Outcomes Study i.e., the Short-Form-36 Health Survey (SF-36) and Short-Form-20 Health Survey (SF-20). However, the Nottingham Health Profile (NHP) was used in one study and the European Organisation for Research and Treatment of Cancer Quality of Life Questionnaire (EORTC QLQ-C30) was used in another. Although the number of domains explored varied from one study to the next, the measuring instruments used have excellent psychometric properties and validity.

The 4 studies associated with the highest quality scores explored only a limited number of potential confounders, namely age [37,38], gender [36,41], and socio-demographic and economic factors [38]. Effects of these confounders are reported in Table 2. The other 3 studies did not investigate potential confounders.

### Quality of life as a secondary outcome measure

Of the 23 studies that evaluated the relationship between multimorbidity or comorbidity and QOL as a secondary outcome measure [43-65], most were done in Europe (9 studies) and the United States (12 studies). As with the main outcome studies, each used a simple count of a limited and varying number of chronic medical conditions to evaluate multimorbidity. While there was generally no attempt to assess or account for the severity of individual conditions, one study used a comorbidity index, the Duke Severity of Illness (DUSOI), for this purpose [48]. Diagnostic information was obtained from chart reviews and clinical evaluations (9 studies), from self-report questionnaires (13 studies), or both sources (1 study). Psychiatric comorbidity was evaluated in 13 studies.

As with the results from the main outcome studies, we found an inverse relationship between the number of medical conditions and the QOL relating to physical domains in all studies. However, the relationship between multimorbidity and QOL relating to psychological or social domains was less clear. Some investigators reported an effect of multimorbidity on these domains in patients with 3 or more diagnoses [54], while others reported no effect [48,55].

As in the main outcome studies, tools from the Medical Outcomes Study, including the SF-36 (17 studies), SF-20 (3 studies), and Short-Form-12 Health Survey (SF-12) (1 study), were used to evaluate QOL in most of these studies. However, the NHP was used in one study and the Quality of Well-Being Scale (QWB), in another. In the majority of studies, all of the QOL domains were explored.

**Table 2:** Synthesis of studies on multimorbidity with quality of life as the main outcome measure

Author (Country)	Design	Score	Population	Multimorbidity	QOL scale	Limitations	Conclusions
Cheng 2003 [36] (United States)	Cross-sectional design	17	Ambulatory, family medicine. n = 316 (55–64 years)	7 diagnoses of chronic conditions obtained by chart review.	Medical Outcomes Study (SF-36). Administered by interviewer.	Definition of multimorbidity was based on simple count of diseases. No assessment of disease severity or use of a healthy group for comparison. No mention of psychiatric comorbidity. Limited to low-income population. Small sample. Age of the sample was limited.	For every SF-36 domain, scores obtained in pregeriatric patients are significantly lower than those obtained in the general population. Lower physical component summary scores (PCS) and mental component summary scores (MCS) are associated with a greater number of chronic diseases, but this association is much stronger for PCS than MCS.
Wensing 2001 [37] (Netherlands)	Cross-sectional design	18	Ambulatory, family medicine. n = 4,112 (18+ years)	25 diagnoses of chronic conditions, with the possibility of including other diagnoses reported spontaneously. Self-administered questionnaire.	Medical Outcomes Study (SF-36); 8 domains. Self-administered.	Definition of multimorbidity was based on simple count of diseases. Medical conditions were self-reported by patient, with no assessment of disease severity. Psychiatric comorbidity was not evaluated. Prevalences of chronic conditions were abnormally low, consistent with a selection or information bias.	The QOL in each of the domains declines with the number of diagnoses (0, 1, 2 and over) but less so for the mental health domain. The QOL score declines with age, especially in physical domains.
Michelson 2001 [38] (Sweden)	Cross-sectional design	16	General adult population, stratified according to age. n = 3,069 (18–79 years)	13 diagnoses of chronic conditions, divided into 4 categories based on the number of problems: (0, 1–2, 3–4, 5+). Self-administered questionnaire.	European Organization for Research and Treatment of Cancer Quality of Life Questionnaire (EORTC QLQ); 5 domains. Self-administered.	Too few diagnoses considered. Medical conditions were self-reported by patients, with no assessment of disease severity. Psychiatric comorbidity was not evaluated. Although adequate for use as a generic measure, the QOL questionnaire was developed for cancer patients.	The presence of multiple chronic problems is associated with a lower QOL score. This association is present for each age group and tends to reduce the relationship between age and QOL. The impact of socio-demographic and economic factors varies with age.
Cuijpers 1999 [39] (Nether-lands)	Cross-sectional design at the beginning of a cohort study	10	Residents of homes for the elderly. n = 211 (Mean = 84.3 years)	7 diagnoses of chronic conditions, with the possibility of including other diagnoses reported spontaneously. Questionnaire administered by the nursing staff.	Short-Form-20 Health Survey (SF-20); 5 domains. Administered by interviewer.	Too few diagnoses considered. No assessment of disease severity. Psychiatric comorbidity was not evaluated. Data collection procedure was not standardized. Many refusals to participate (30%), including some for health reasons. Small sample. Aged patients.	A lower QOL score is associated with a high number of chronic conditions.
Grimby and Svanborg 1997 [40] (Sweden)	Cross-sectional design in a cohort follow-up	14	General ambulatory. n = 565 (76 years)	16 diagnoses of chronic conditions present in > 5%. Medical questionnaire.	Modified Nottingham Health Profile (NHP); part I: 6 dimensions; part II: 5 questions. Self-administered.	Definition of multimorbidity was based on a simple count of diseases. No assessment of disease severity. Health of nonrespondents was not comparable (more ill). No age variation (76 years).	The loss of QOL is proportional to the number of diagnoses for the dimensions of energy, pain, mobility, and sleep. For social and emotional dimensions, QOL is little influenced until health is significantly impaired (4 or more diagnoses).
Kempen 1997 [41] (Nether-lands)	Cross-sectional design at the beginning of a cohort study	17	Ambulatory, family medicine. n = 5,279 (57+ years)	18 diagnoses of chronic conditions. Questionnaire administered by interviewer.	Short-Form-20 Health Survey (SF-20); 6 domains. Administered by interviewer or self-administered.	Definition of multimorbidity was based on simple count of diseases reported by the patient. Use of a list of diagnoses in correlation and multiple regression analyses. No assessment of disease severity or psychiatric comorbidity. Age of the sample was limited.	The presence of chronic medical conditions explains a high proportion of the variance (25%) in the QOL score in most domains, especially self-perceived health. Personality influences QOL scores, especially in the mental health domain. The association between the number of chronic conditions and the QOL score is slightly stronger for women than men.
Fryback 1993 [42] (United States)	Cross-sectional design	13	General ambulatory. n = 1,356 (45–89 years)	28 diagnoses of chronic conditions, with the possibility of including other diagnoses reported spontaneously. Questionnaire administered by interviewer.	Medical Outcomes Study (SF-36) reduced to 2 domains. Quality of Well-Being scale (QWB). Administered by interviewer.	Definition of multimorbidity was based on a simple count of diseases reported by patient. No assessment of disease severity. QOL questionnaire completed by the same interviewer immediately after the medical questionnaire. Characteristics of the healthy group were not described. Multimorbidity data were not adjusted for age. Questionnaire did not include all domains traditionally included in QOL assessment.	The QOL score, as estimated with all of the measuring instruments, decreases with the number of chronic medical conditions. However, only limited domains of QOL were evaluated.

QOL: Quality of life

## Discussion

Although this systematic review confirms the inverse relationship between multimorbidity and QOL, it also raises some important questions. First, the relative lack of studies in primary care evaluating the association between multimorbidity and QOL or HRQOL is surprising given the number of patients who suffer from multiple concurrent chronic conditions. Although the existence of this association makes logical sense, it still has to be demonstrated and thoroughly studied to find ways of improving care for specially affected patients. Thus, the pressing question may not be whether there is an association but rather how strong is the association and what factors are responsible for it? Identifying these factors may contribute to better care for the affected patients. There is a clear need for further studies to address these issues.

Ultimately, multimorbidity has the potential to affect all domains of QOL. However, the influence of multimorbidity on the social and psychological dimensions of QOL is much less clear than its influence on the physical domains. It is noteworthy that several studies showed a significant decline in social and psychological dimensions of QOL in patients with 3, 4, or more concurrent diagnoses. What does this finding mean? Is there any bias that can explain this difference, or is it related to a certain capacity for adaptation? Are there other factors associated with this finding? All of these questions have yet to be answered.

All the studies examined were cross-sectional in nature. The effect of multimorbidity may vary over time. Some medical conditions may improve while others worsen resulting in various effects on QOL. Therefore, cross-sectional studies may not capture the real effect of multimobidity on QOL and predict the direction of change over time.

### Defining and measuring multimorbidity

The absence of a uniform way of defining and measuring multimorbidity is of special concern and may explain some of the variability in our results. In most of the studies we evaluated, investigators had used only a simple list of diseases to identify concurrent medical conditions in patients, providing very incomplete information. Furthermore, the numbers and types of medical conditions in these lists varied among the studies, precluding comparisons.

Given the urgent need for conceptual clarity, Van den Akker and colleagues' definition of multimorbidity should be refined and advanced to achieve a common understanding. A distinction must be made between simple and complex chronic diseases. Treated hypothyroidism (simple) and ischemic heart disease (complex) obviously do not have the same impact on QOL. Moreover, the influence of single-organ versus multi-organ diseases needs to be appropriately weighed. Additional factors to be considered when defining multimorbidity include the severity of the conditions and the presence or absence of associated pain.

The use of self-reported diagnoses in many studies is another methodological limitation that may have introduced error. Patients may confuse symptoms and minor ailments with more significant disease states or may forget to report important diagnoses that are still active. Self-reporting may even be completely inaccurate in the presence of psychosomatic disorders. Conducting a chart review, clinical interview or using any specific standardized method may be a better way to obtain data related to diagnoses.

Another methodological limitation of most of the studies evaluated was their failure to consider the influence of psychiatric comorbidity. This was either because psychiatric diagnoses were not included in the lists of disease states or because patients presenting with psychiatric diagnoses were excluded from QOL assessment. Given the importance of psychiatric conditions in primary care practice with a prevalence of more than 20% [66], this limitation is simply unacceptable.

### Confounders

QOL tends to decrease with age [67], whereas the number of diagnoses increases with age. Thus, it is appropriate to consider age as a potential confounding variable. The effect of age was explored in some of the studies that used QOL as a main outcome measure [37,38,41]. Reference to established norms would have facilitated interpretation of these results.

Only a few of the studies evaluated had explored the effect of gender. Furthermore, their results were contradictory, with gender being more detrimental to the QOL of women in some cases [41,58] and men, in others [51].

Little has been reported about the effects of other potential confounding variables (e.g., socio-demographic and economic data, health habits, social support, number of drugs prescribed), although these factors are recognized as having an impact on QOL [68-71]. A few of the studies that used QOL as a secondary outcome measure considered the influence of socio-economic variables; however, their results were ambiguous, showing an impact in only about half of the studies. Some studies also demonstrated that, although socio-economic variables and health habits were significant predictors of QOL, the number of comorbidities was the strongest independent predictor of QOL [41,56]. Only one study took into account social support, and this study revealed a relationship with the mental dimension of QOL [58]. Only one study took into account the number of drugs prescribed and found an impact on the physical domain of QOL [49]. This study looked specifically at comorbidities associated with arterial hypertension and their impact on QOL. Finally, other potential confounding variables such as marital status and living arrangements were considered in some studies, with demonstration of an impact on QOL in about half the studies.

Many other factors should be explored in this regard. For example, the presence of coexisting *acute *conditions, the time since the diagnosis of important chronic conditions, and the duration and prognosis of health problems are among factors that may explain some of the variability in QOL or HRQOL.

### Research agenda

In light of the findings of this systematic review, further research is needed to clarify the relationship between multimorbidity and QOL. The early work will certainly be conceptual and theoretical. The resultant conceptual clarity would benefit both researchers and practitioners. How do we define and how should we measure multimorbidity are among the first questions to be addressed. More descriptive studies, which take into account the influence of multiple potential confounders, can then be conducted. Multivariate analyses will help control for the effects of these confounding variables. The effects of age and gender also need to be further explored, with reference to established norms. Although there is still a need for cross-sectional studies, longitudinal studies are also needed to identify changes in the relationship between multimorbidity and QOL over time.

### Study limitations

The main limitation of a systematic review is its inability to include all of the relevant literature. We realize that some articles may have been missed during the search stage. However, our review of a huge number of abstracts generated by different strategies improved the sensitivity of the search. Obviously, the absence of a keyword for multimorbidity is a limitation. However, we found that in the majority of cases in which the term "multimorbidity" was used to search, the term "comorbidity" also appeared in the list of keywords. Adding the term "chronic disease" also helped to circumvent the problem. Restricting the search to articles published in French or English is another limitation.

## Conclusion

This systematic review focused on the relationship between the presence of several chronic coexisting medical conditions and QOL or HRQOL in a primary care setting. However, the studies evaluated had important limitations due to the lack of a uniform definition for multimorbidity or comorbidity, the absence of assessment of disease severity, the use of self-reported diagnoses, and the frequent exclusion of psychiatric diagnoses. The potential impact of important confounding variables was also neglected. In light of these observations, it seems clear that further studies are needed to clarify the impact of multimorbidity on QOL or HRQOL and its various dimensions (i.e., physical, social and psychological). A clear understanding of this relationship will ultimately help both researchers and primary health care professionals to deliver more comprehensive care.

## Author contributions

MF was responsible for the conception and design of this systematic review and was also involved in the literature review. In addition, he was responsible for critically assessing the evaluated articles and drafting this manuscript. He takes responsibility for the integrity of the work as a whole and provided final approval of this version of the manuscript.

LL provided a major contribution to the literature review and critical appraisal of the identified articles. She also participated in the drafting of this manuscript and gave final approval of this version.

CH participated in both the conception and design of this review. She also contributed by critically revising this manuscript and gave final approval of this version.

AV participated in the design of this review. He also made an important contribution in critically revising the manuscript and gave final approval of this version.

ALN participated in the drafting of the manuscript and made an important contribution by critically revising it. He also gave final approval of this version.

DM participated in the drafting of the manuscript and made an important contribution by critically revising it. She also gave final approval of this version.
